# Supplementary figures and images for: Septin 7 interacts with Numb to preserve sarcomere structural organization and muscle contractile function
Source: eLife. 2024 May 2;12:RP89424. doi: 10.7554/eLife.89424 (PMC11065422; doi:10.7554/eLife.89424)

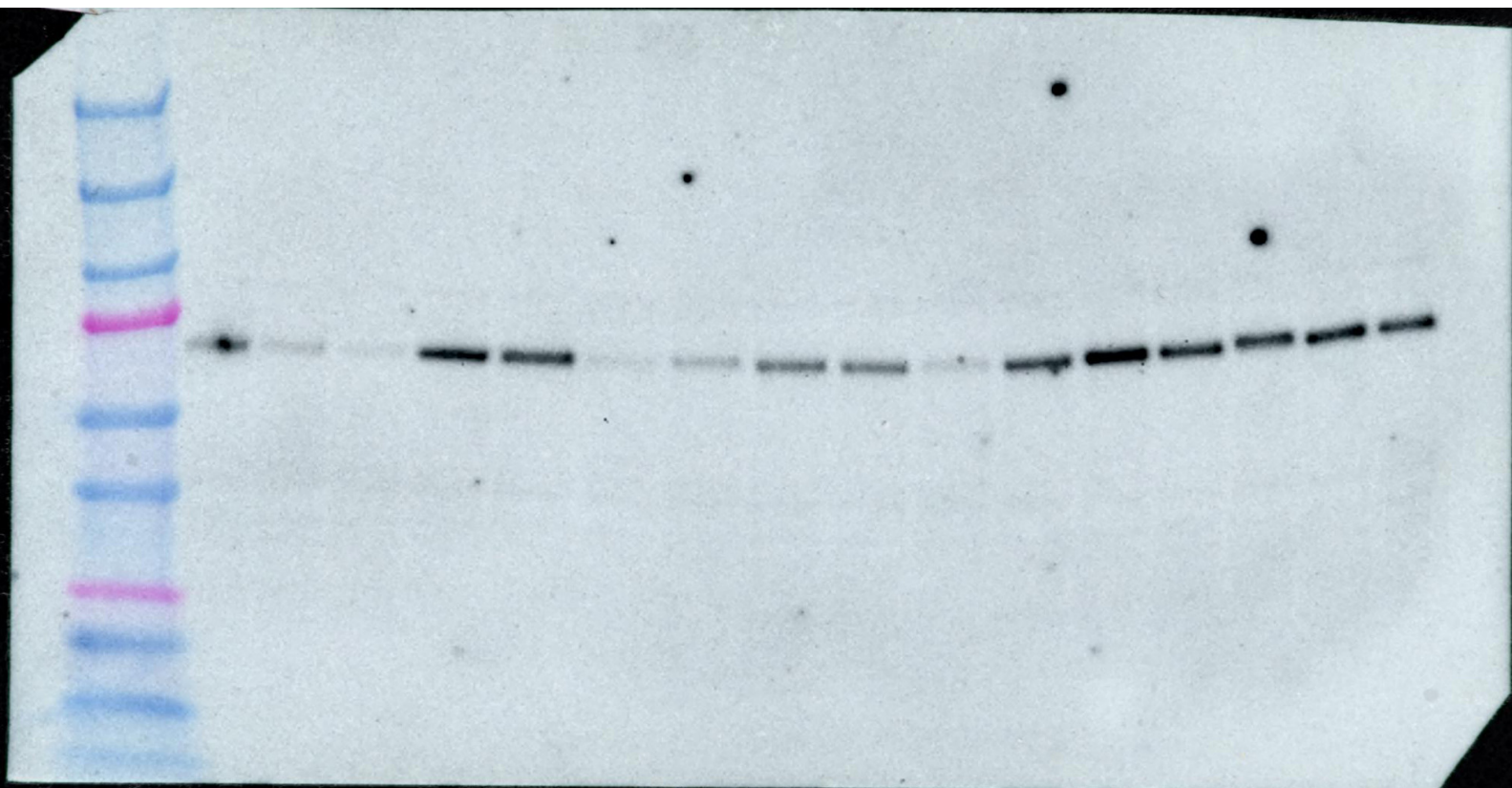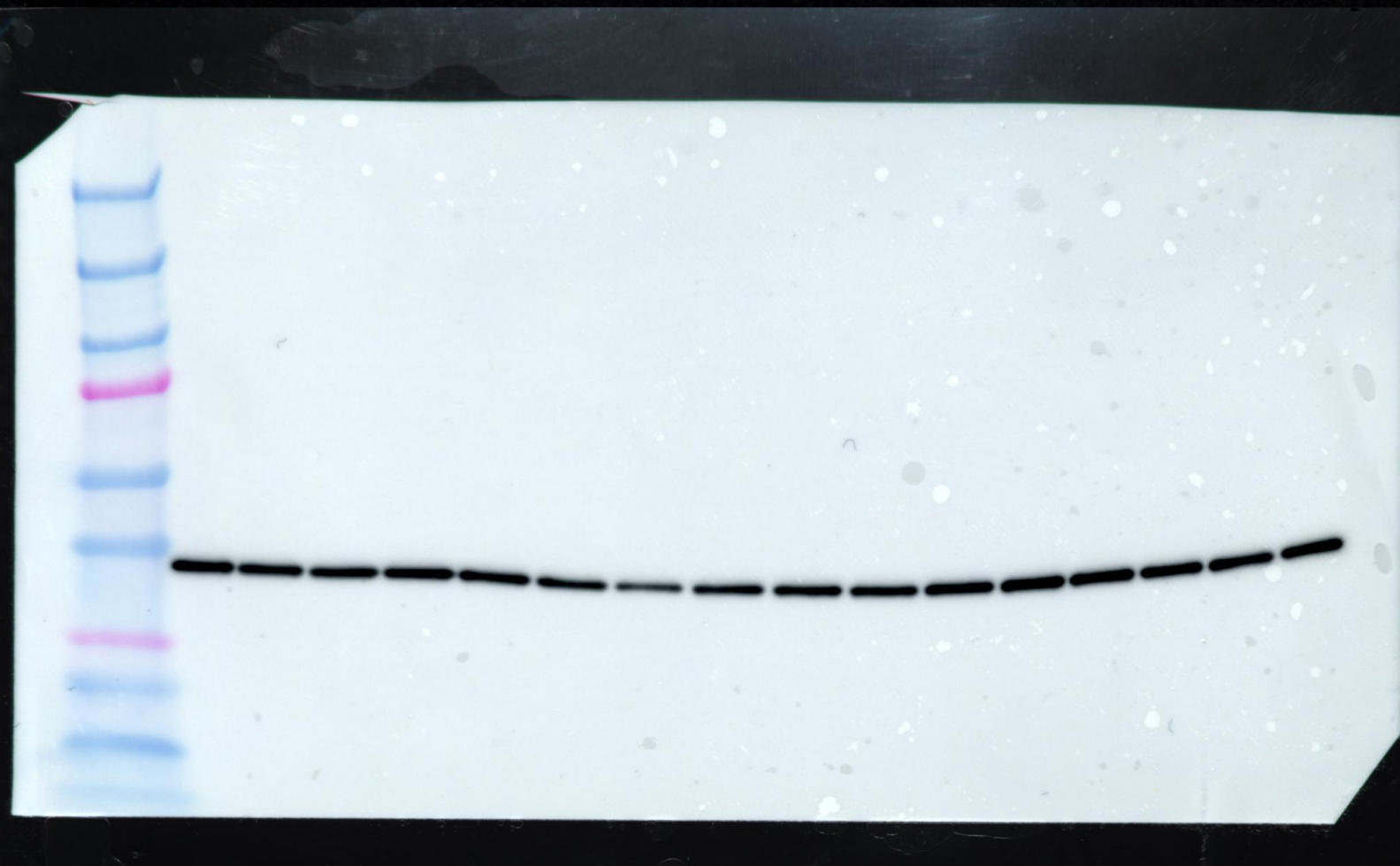

Supplement: Figure 2—source data 1. [file elife-89424-fig2-data1.pdf]

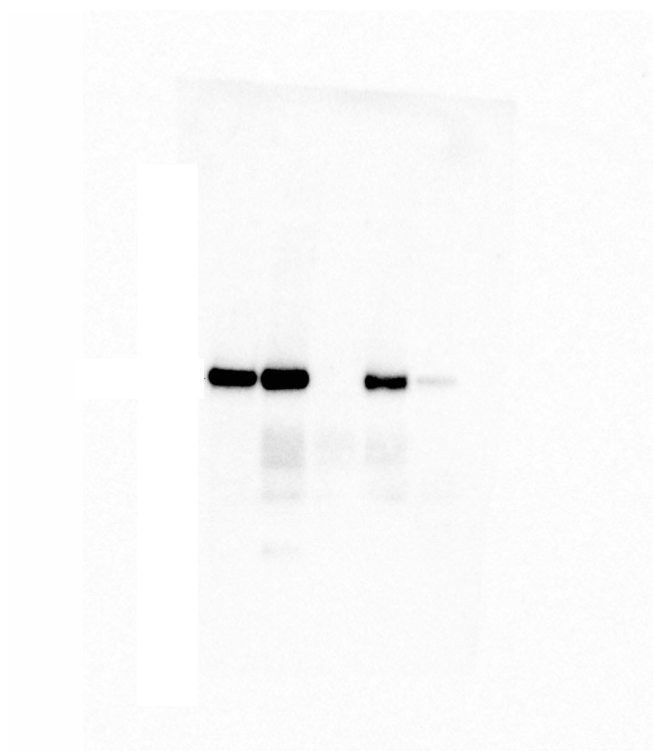

Supplement: Figure 5—source data 1. [file elife-89424-fig5-data1.pdf]

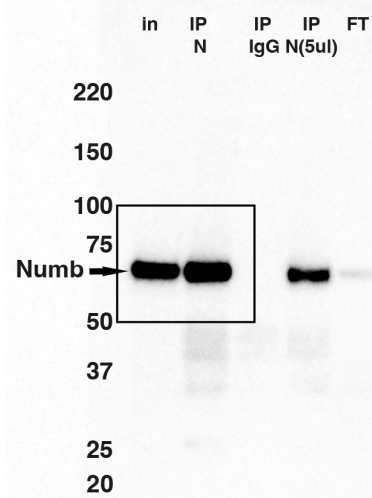

Supplement: Figure 5—source data 2. [file elife-89424-fig5-data2.pdf]

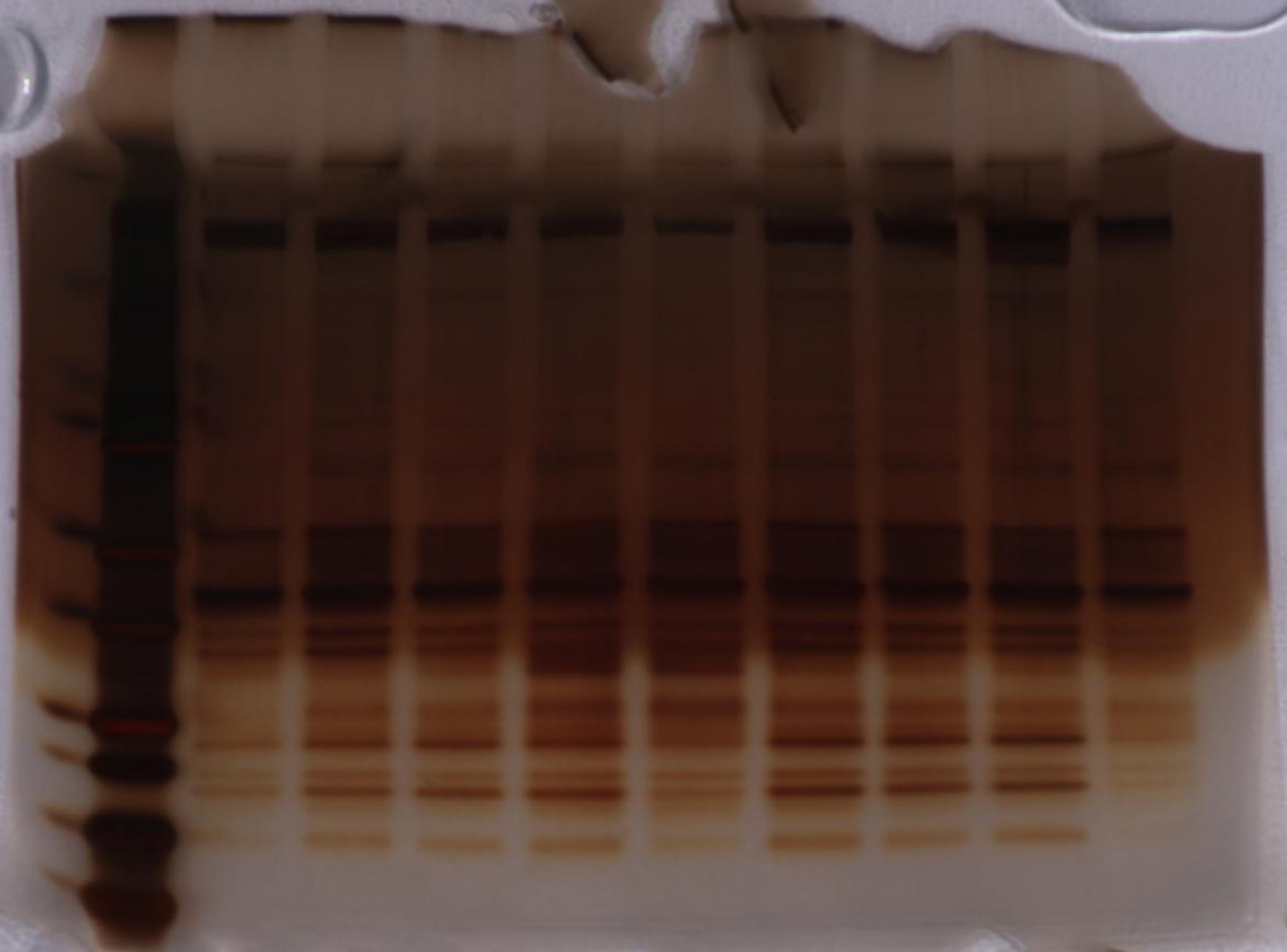

Supplement: Figure 5—source data 3. [file elife-89424-fig5-data3.pdf]

# IP Silver stain

**a**

**b**

**c**

**d**

**e**

**f**

**g**

250

150

100

75

50

37

25

20

15

10

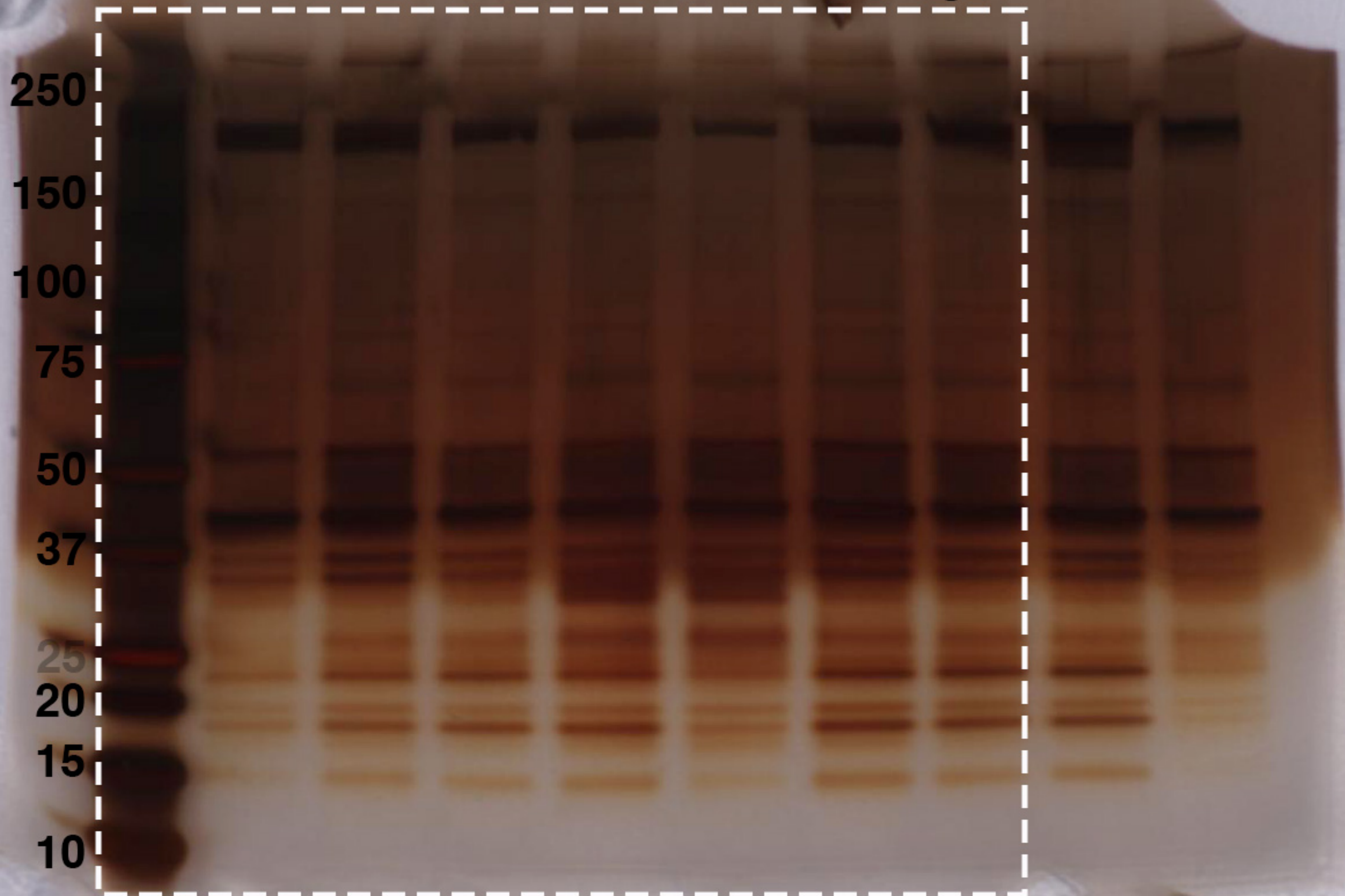

Supplement: Figure 5—source data 4. [file elife-89424-fig5-data4.pdf]

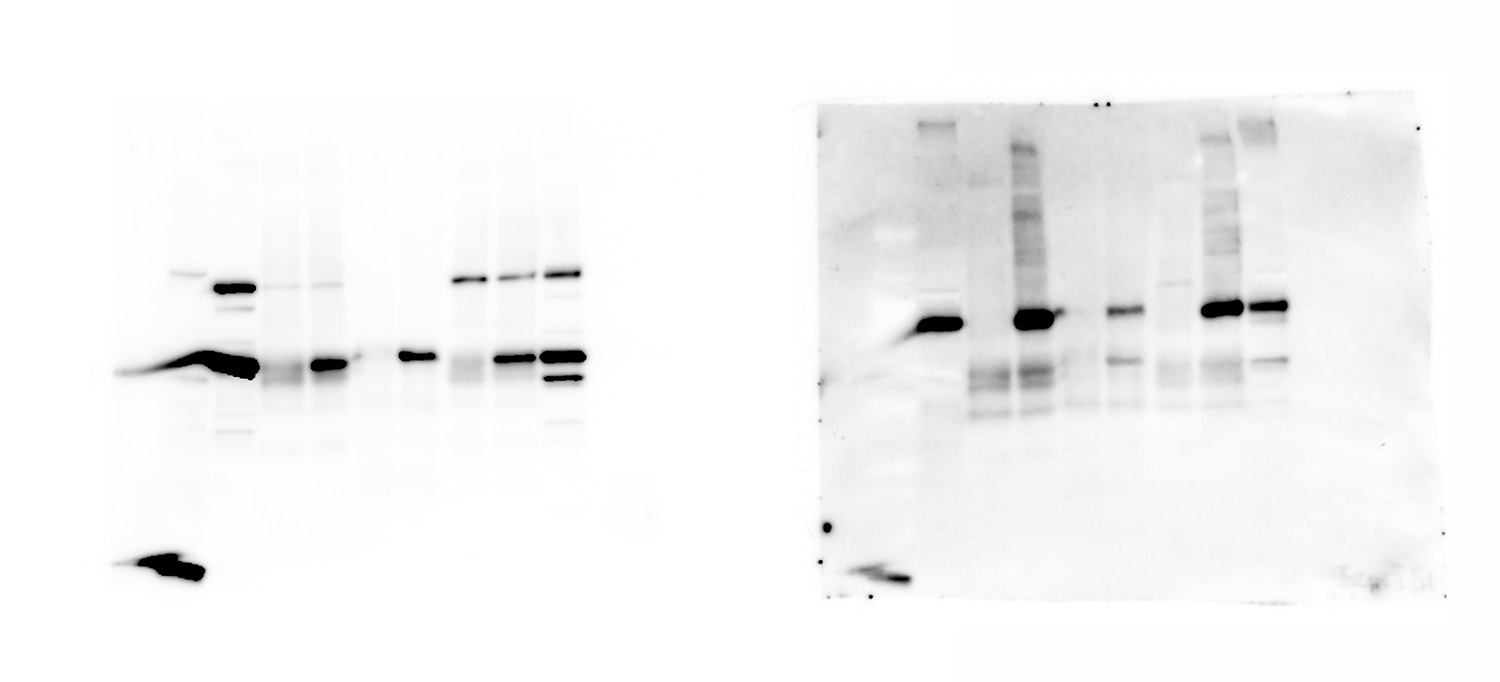

Supplement: Figure 6—source data 1. [file elife-89424-fig6-data1.jpg]

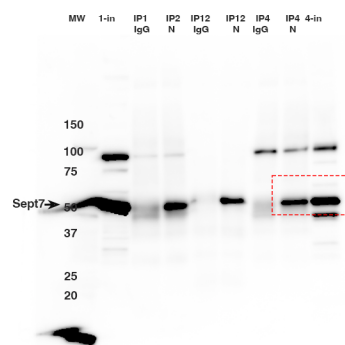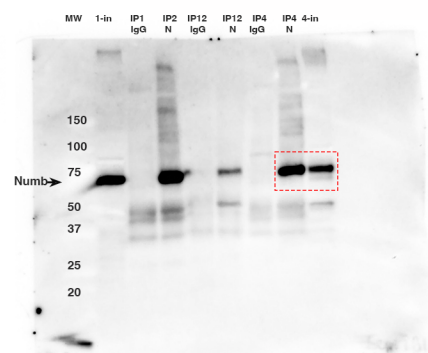

Supplement: Figure 6—source data 2. [file elife-89424-fig6-data2.pdf]
