# Supplementary material for: Septin 7 interacts with Numb to preserve sarcomere structural organization and muscle contractile function
Source: eLife. 2024 May 2;12:RP89424. doi: 10.7554/eLife.89424 (PMC11065422; doi:10.7554/eLife.89424)
Supplement: Figure 2—source data 2. [file elife-89424-fig2-data2.pdf]

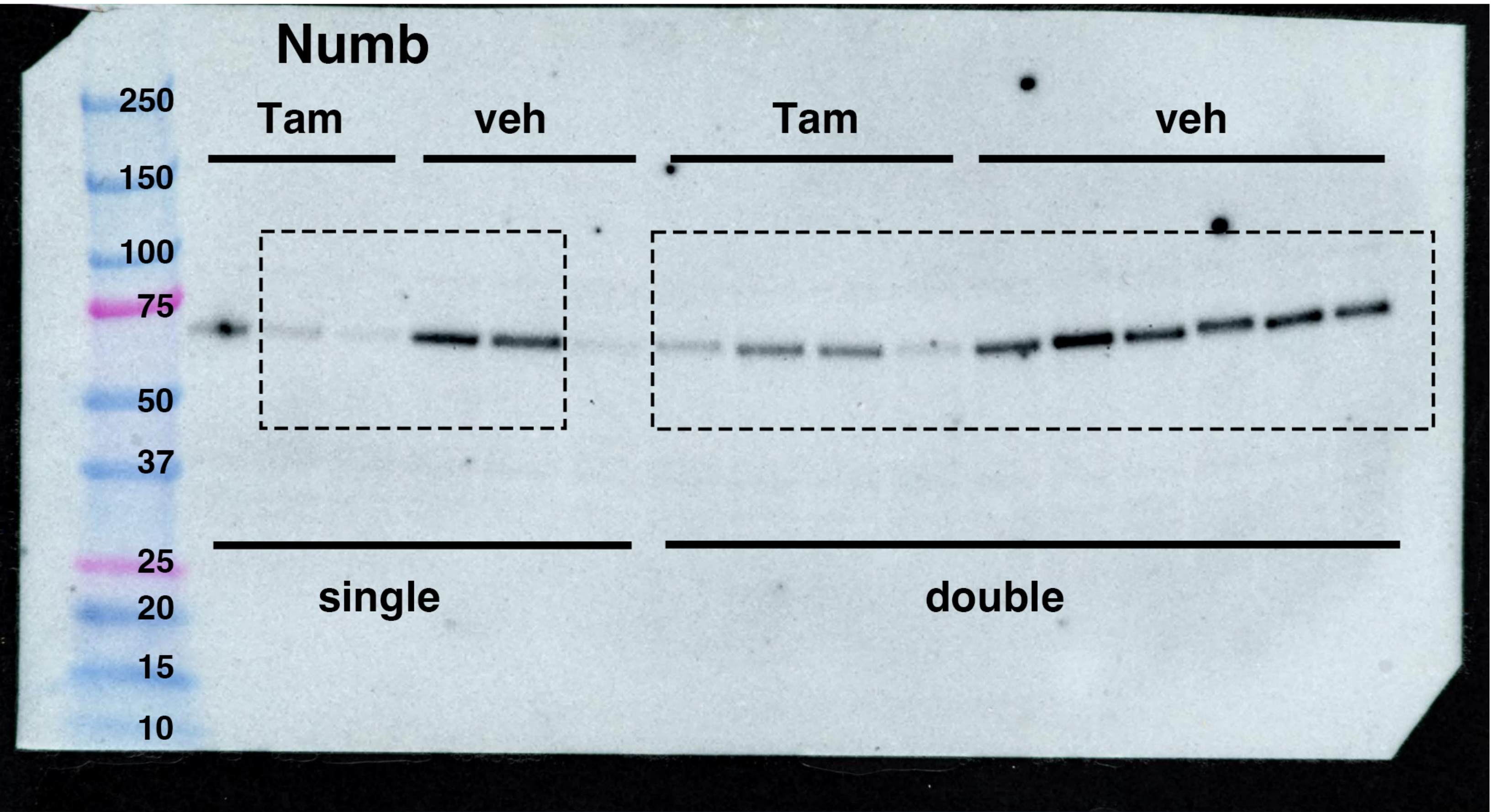
Numb
250
150
100
75
50
37
25
20
15
10
Tam
veh
Tam
veh
single
double

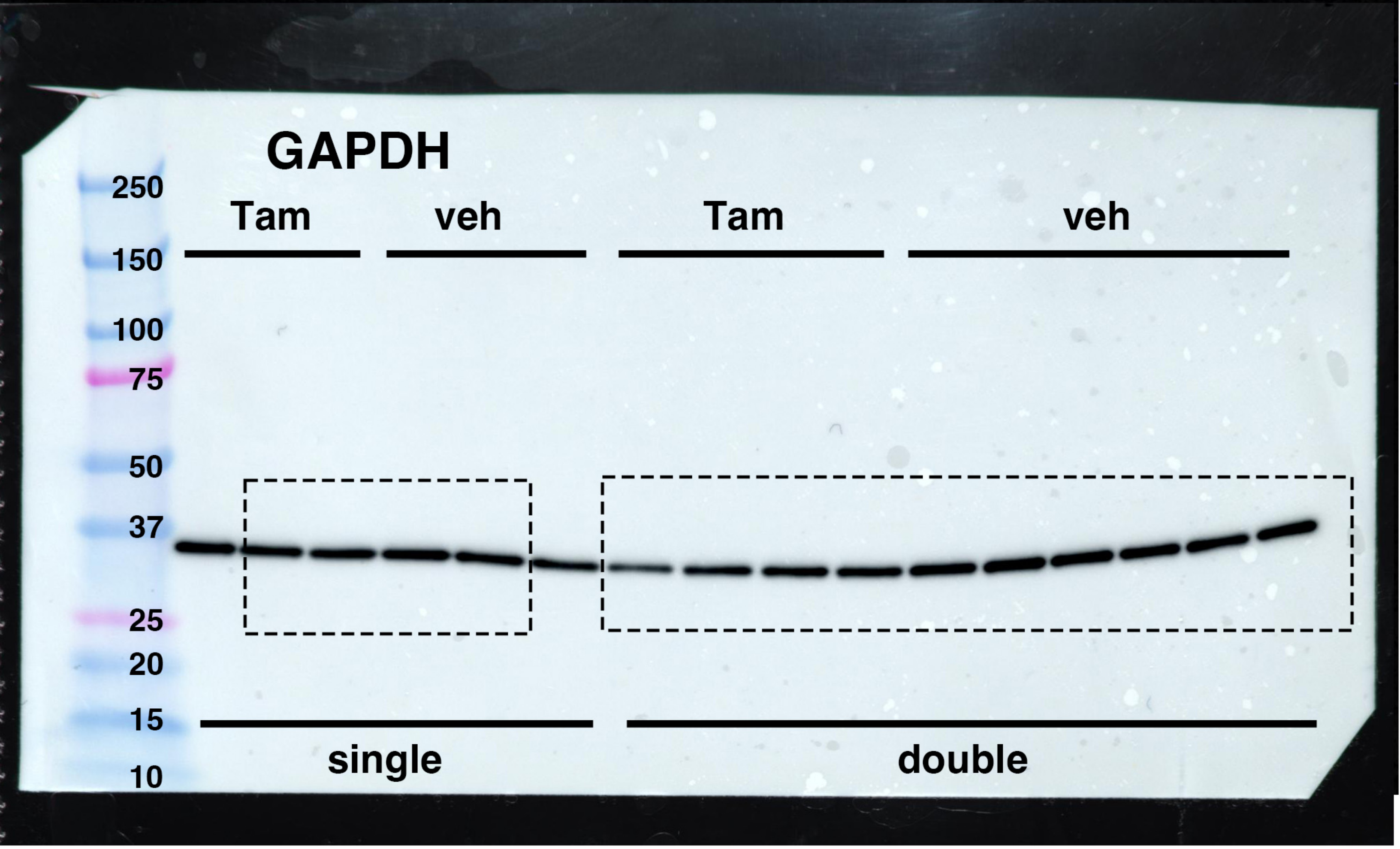
GAPDH
250
150
100
75
50
37
25
20
15
10
Tam
veh
Tam
veh
single
double
