# Supplementary material for: Septin 7 interacts with Numb to preserve sarcomere structural organization and muscle contractile function
Source: eLife. 2024 May 2;12:RP89424. doi: 10.7554/eLife.89424 (PMC11065422; doi:10.7554/eLife.89424)
Supplement: Table 1—source data 1. [file elife-89424-table1-data1.pdf]

O55131|SEPT7_MOUSE Septin-7

Example Peptide Abundances for VNIIPLIAK (2+)

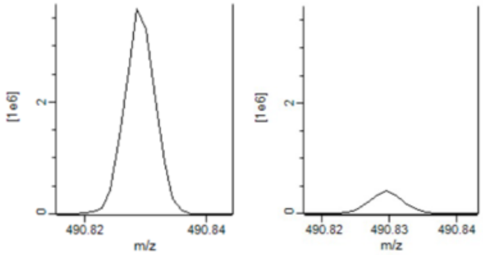

67 - Numb   67 - Control

Peptides	Sequence coverage [%]	Protein Score	Abundance Ratio (Numb/ Control)	P-value (Control vs. Numb)
32	67.7	210	82.9	3.6E-03

Example MS/MS Spectra

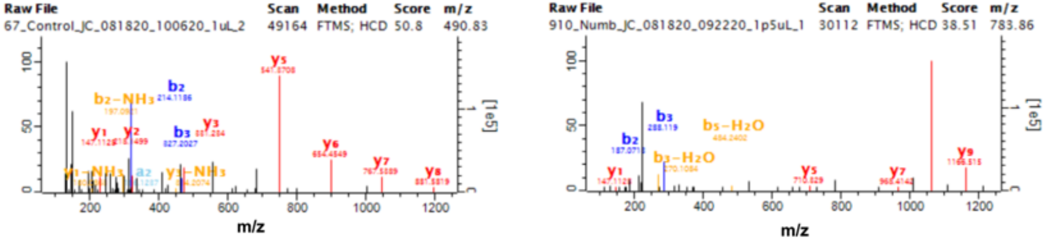

VNIIPLIAK (2+)   ADTLTPEECQQFK (2+)
