# Supplementary material for: Septin 7 interacts with Numb to preserve sarcomere structural organization and muscle contractile function
Source: eLife. 2024 May 2;12:RP89424. doi: 10.7554/eLife.89424 (PMC11065422; doi:10.7554/eLife.89424)
Supplement: Table 1—source data 2. [file elife-89424-table1-data2.pdf]

Q01063|PDE4D_MOUSE cAMP-specific 3,5-cyclic phosphodiesterase 4D

Example Peptide Abundances for ELTHLSEMSR (3+)

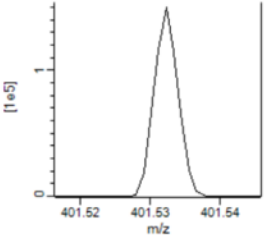

520 - Numb

No Signal

Control

Peptides	Sequence coverage [%]	Protein Score	Abundance Ratio (Numb/ Control)	P-value (Control vs. Numb)
5	9.6	14	1.0E+06	0

Example MS/MS Spectra

Raw File	Scan	Method	Score	m/z
520_Numb_JC_081820_100720_4uL_2	21835	FTMS; HCD	52.07	401.53

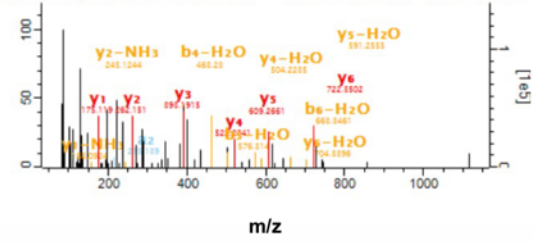

ELTHLSEMSR (3+)

Raw File	Scan	Method	Score	m/z
1415_Numb_JC_081820_092220_5uL_1	36165	FTMS; HCD	59.61	689.35

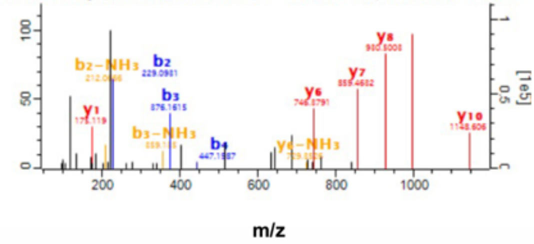

NNFAALTNLQDR (2+)
