# Supplementary material for: Septin 7 interacts with Numb to preserve sarcomere structural organization and muscle contractile function
Source: eLife. 2024 May 2;12:RP89424. doi: 10.7554/eLife.89424 (PMC11065422; doi:10.7554/eLife.89424)
Supplement: Table 1—source data 3. [file elife-89424-table1-data3.pdf]

Q80YV2|NIPA_MOUSE Nuclear-interacting partner of ALK

Example Peptide Abundances for AVLTILLAHKR (3+)

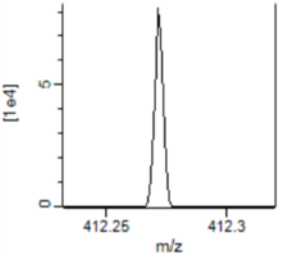

1617 - Numb

No Signal

Control

Peptides	Sequence coverage [%]	Protein Score	Abundance Ratio (Numb/ Control)	P-value (Control vs. Numb)
17	47.1	85	1.0E+06	0

Example MS/MS Spectra

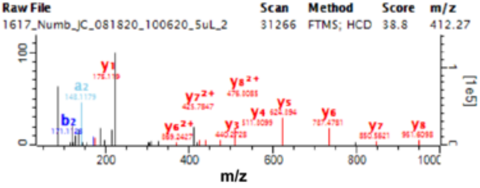

AVLTILLAHKR (3+)

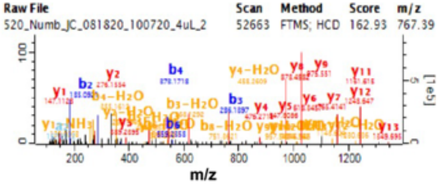

AATSEGPLFAASIEK (2+)
