# Supplementary material for: Septin 7 interacts with Numb to preserve sarcomere structural organization and muscle contractile function
Source: eLife. 2024 May 2;12:RP89424. doi: 10.7554/eLife.89424 (PMC11065422; doi:10.7554/eLife.89424)

Q8K2H6|APC10_MOUSE Anaphase-promoting complex subunit 10

Example Peptide Abundances for VGNNFHNLQEIR (3+)

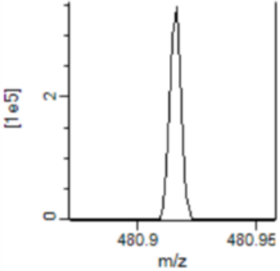

No Signal

Peptides	Sequence coverage [%]	Protein Score	Abundance Ratio (Numb/ Control)	P-value (Control vs. Numb)
4	31.4	11	1.0E+06	0

520 - Numb

Control

Example MS/MS Spectra

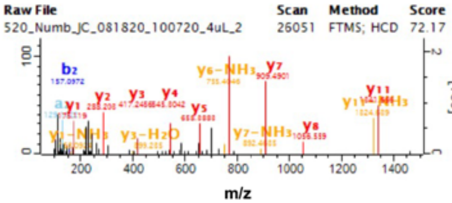

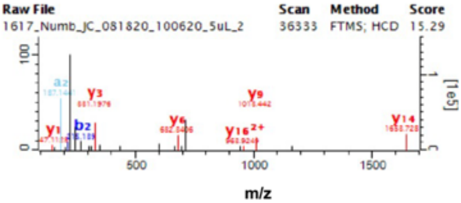

Supplement: Table 1—source data 6. [file elife-89424-table1-data6.pdf]
